# Supplementary material for: The Sub-Nuclear Localization of RNA-Binding Proteins in KSHV-Infected Cells
Source: Cells. 2020 Aug 25;9(9):1958. doi: 10.3390/cells9091958 (PMC7564026; doi:10.3390/cells9091958)
Supplement: Supplementary file 1 [file cells-09-01958-s001.zip › Supplemental material/Supplementary figures Alkalay et al revised clean.pdf]

Supplemental figures and movies

The sub-nuclear localization of RNA-binding proteins in KSHV-infected cells

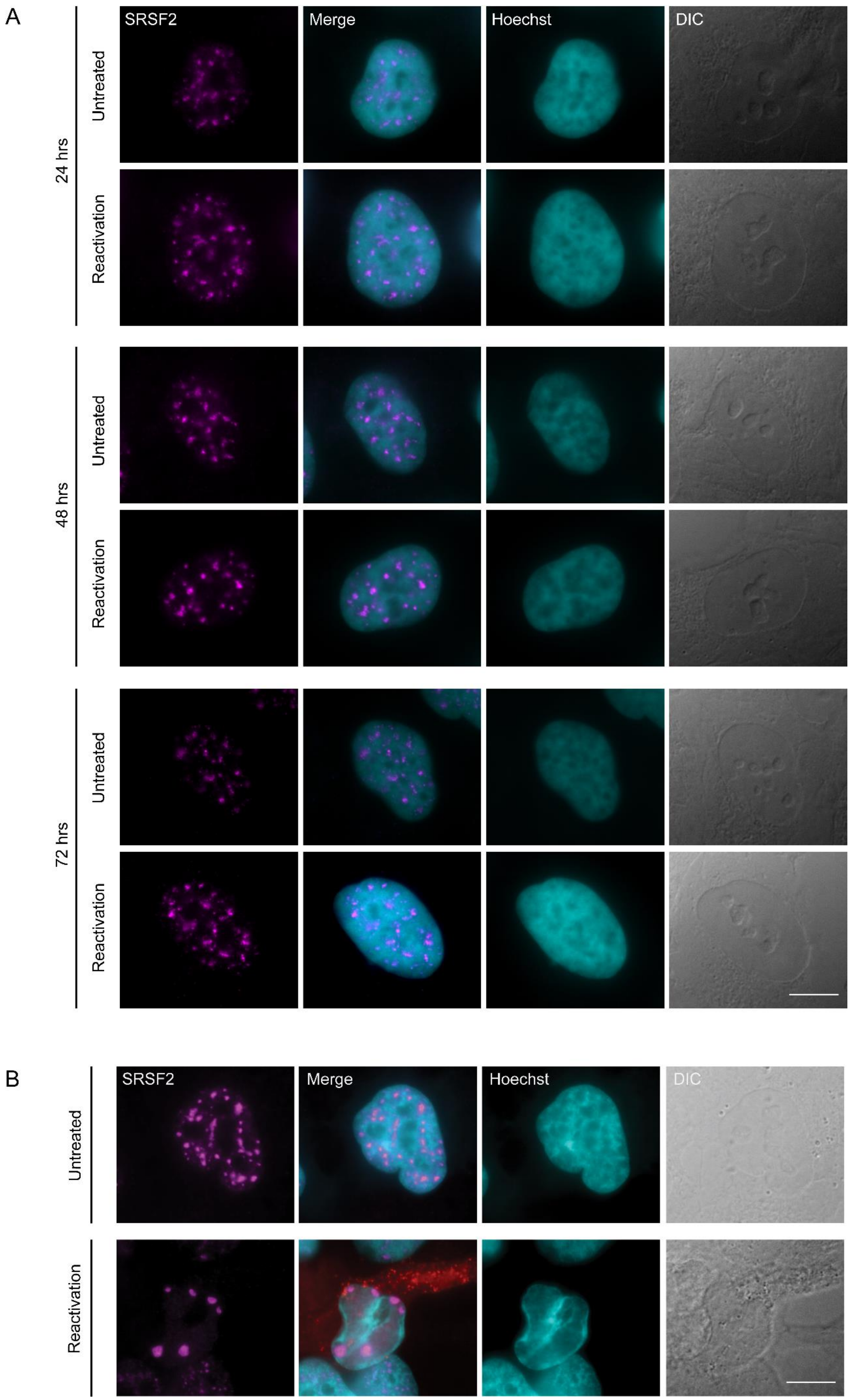
A
SRSF2
Merge
Hoechst
DIC
24 hrs
48 hrs
72 hrs
Untreated
Reactivation
B
SRSF2
Merge
Hoechst
DIC
Untreated
Reactivation

Figure S1. Treatment with n-Butyrate and Doxycycline does not induce changes in chromatin organization and SRSF2 distribution. Un-infected SLK cells were treated with Dox and n-Butyrate for 24, 48 and 72 hrs. No changes in chromatin localization and nuclear speckle (SRSF2, magenta) distribution were observed. (**B**) BAC16-mCherry-ORF45-iSLK infected cells were induced to undergo lytic reactivation for 72 hrs, as described in Figure S1A. Untreated infected iSLK cells were used as a control (top row). The viral lytic protein ORF45 fused to mCherry (red in the merge), was used as a marker for lytic reactivation, along with SRSF2 staining (magenta) and Hoechst (cyan) staining (bottom row). Bar, 10 µm.

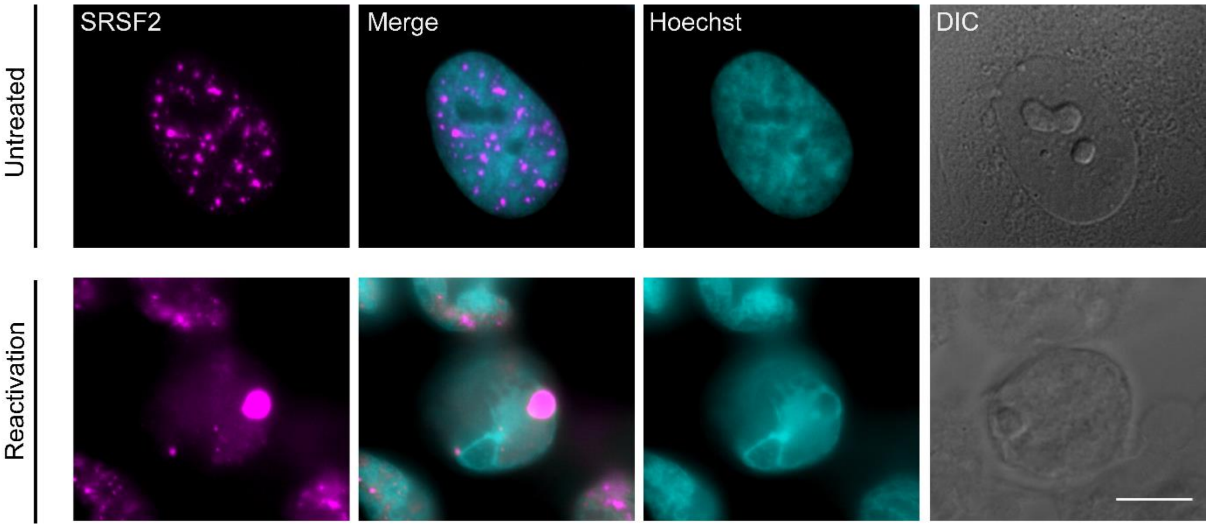

Figure S2. The changes observed for nuclear speckles after inducing for 48 hrs of lytic KSHV infection. (**A**) Infected iSLK cells were treated to induce the lytic KSHV infection cycle for 48 hrs and stained with anti-SRSF2 (magenta; Hoechst in cyan; DIC in grey) Bar, 10 µm.

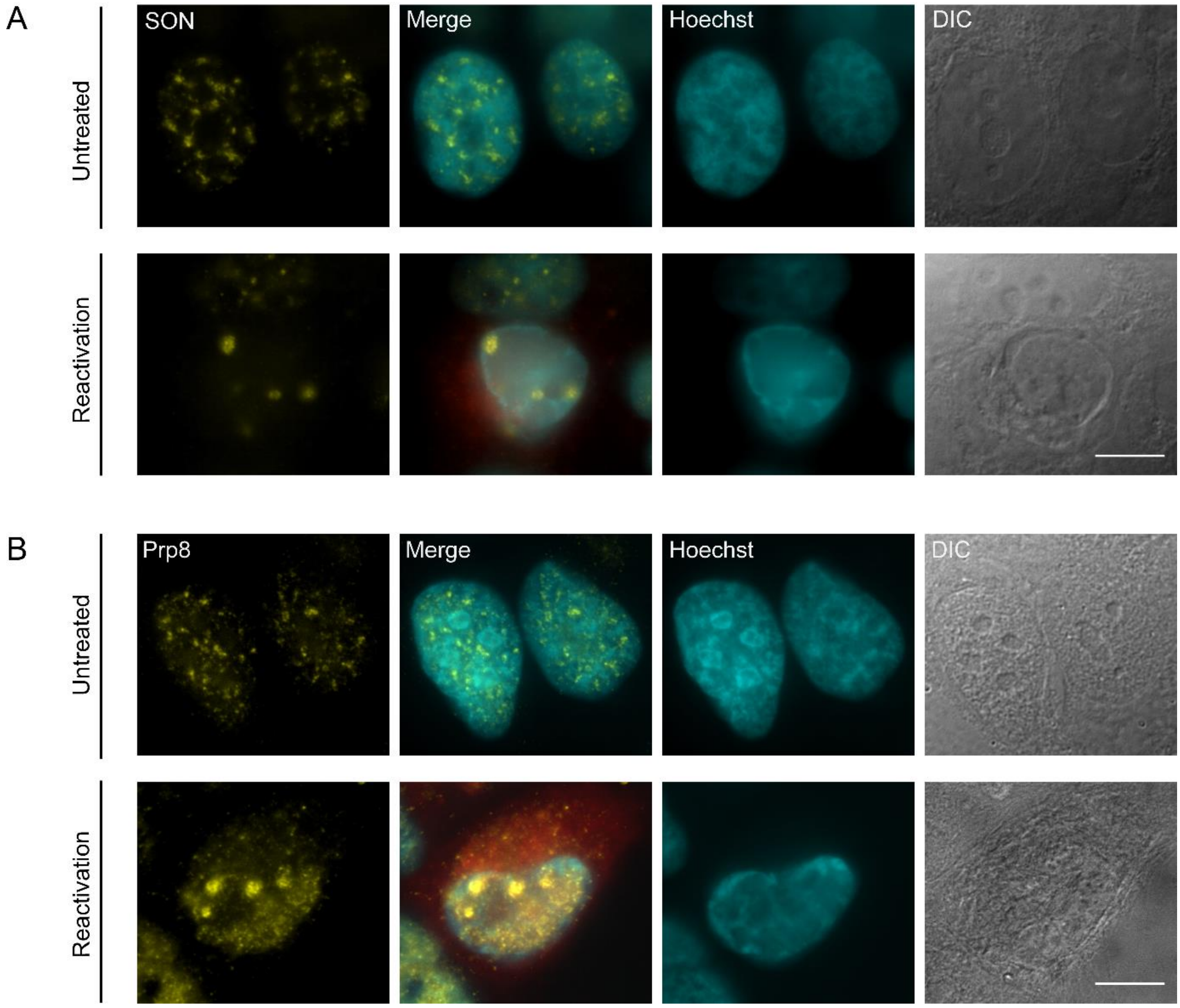

Figure S3. Distribution of SON and Prp8 during lytic reactivation of KSHV infection. Infected iSLK mCherryORF45 cells were treated with n-Butyrate and Doxycycline for 48 hrs to induce the lytic cycle of KSHV infection and stained with (**A**) anti-SON or (**B**) Prp8 (yellow); ORF45 in red in the merge; Hoechst (cyan); DIC in grey. Top row – untreated cells, bottom row – reactivated cells. Bar, 10 µm.

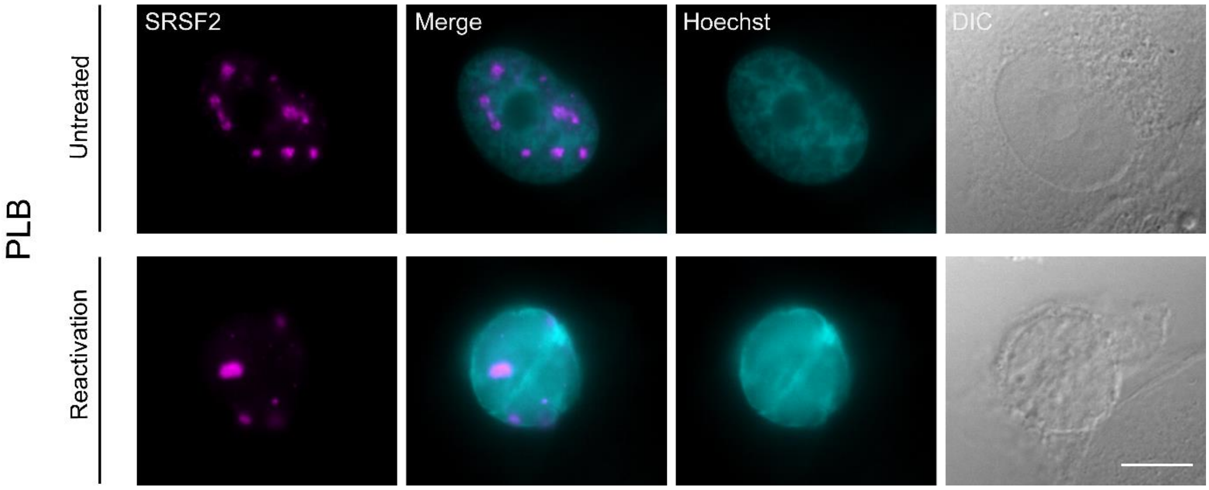

Figure S4. The distribution of nuclear speckles in splicing inhibited cells versus KSHV infected cells. Cells were treated with Pladienolide B (PLB) for 48 hrs. Top – splicing inhibition in control cells; bottom - splicing inhibition in BAC16- infected iSLK cells that were treated to induce the lytic cycle of KSHV infection for 48 hrs and stained with anti-SRSF2 (magenta); Hoechst in cyan; DIC in grey. Bar, 10 µm.

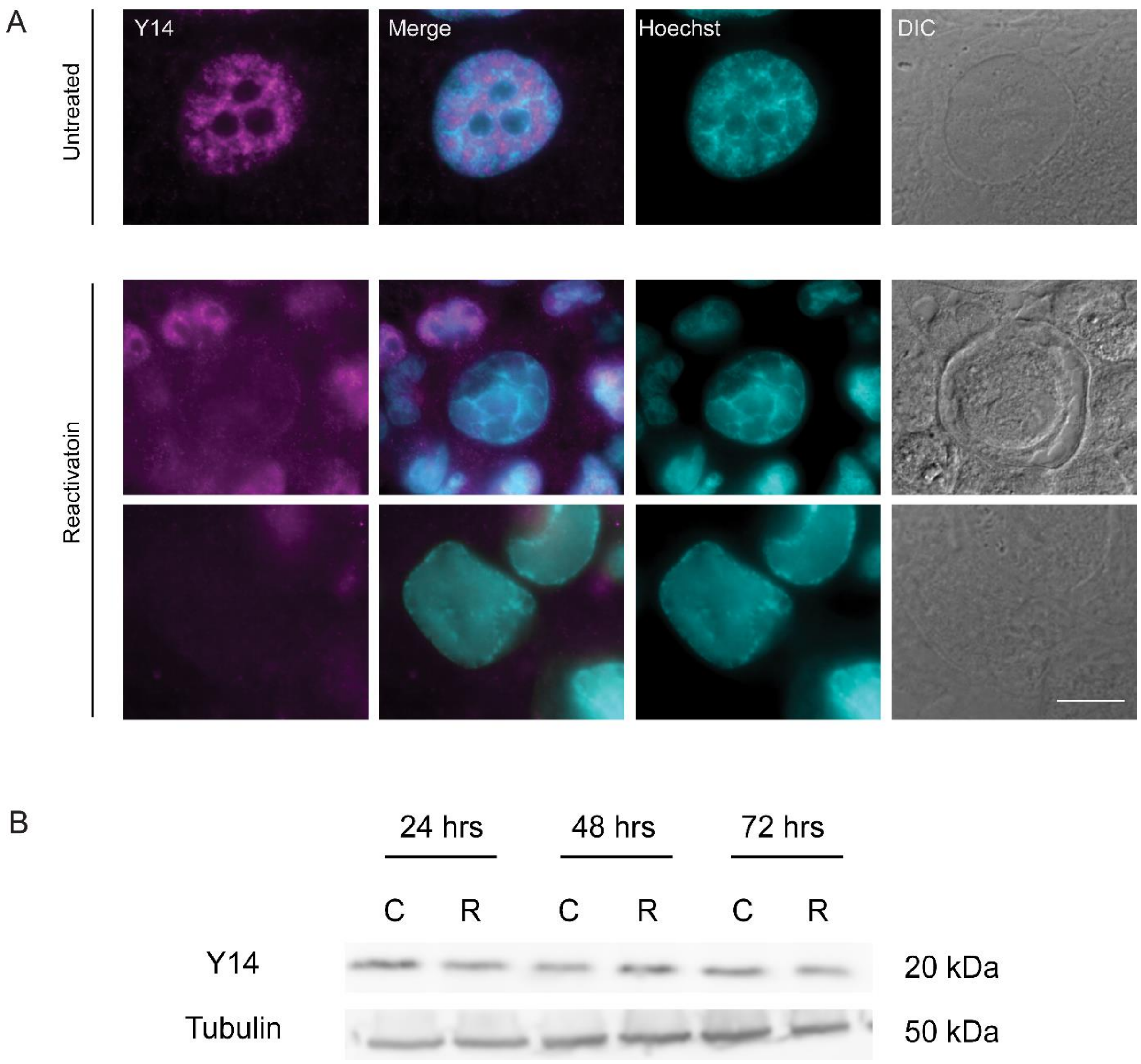

Figure S5. The distribution of Y14 during lytic reactivation of KSHV infection. (**A**) BAC16 -mCherry-ORF45-infected iSLK cells were either left untreated or treated to induce lytic cycle reactivation of KSHV infection for 48 hrs and stained with anti-Y14 (magenta); Hoechst in cyan; DIC in grey. Bar, 10 µm. (**B**) Western blot of Y14 protein levels in cell extracts from control untreated (C) and reactivated (R) cells for the indicated time points. Tubulin was used as a loading control. Gel is a representative of 3 different experiments.

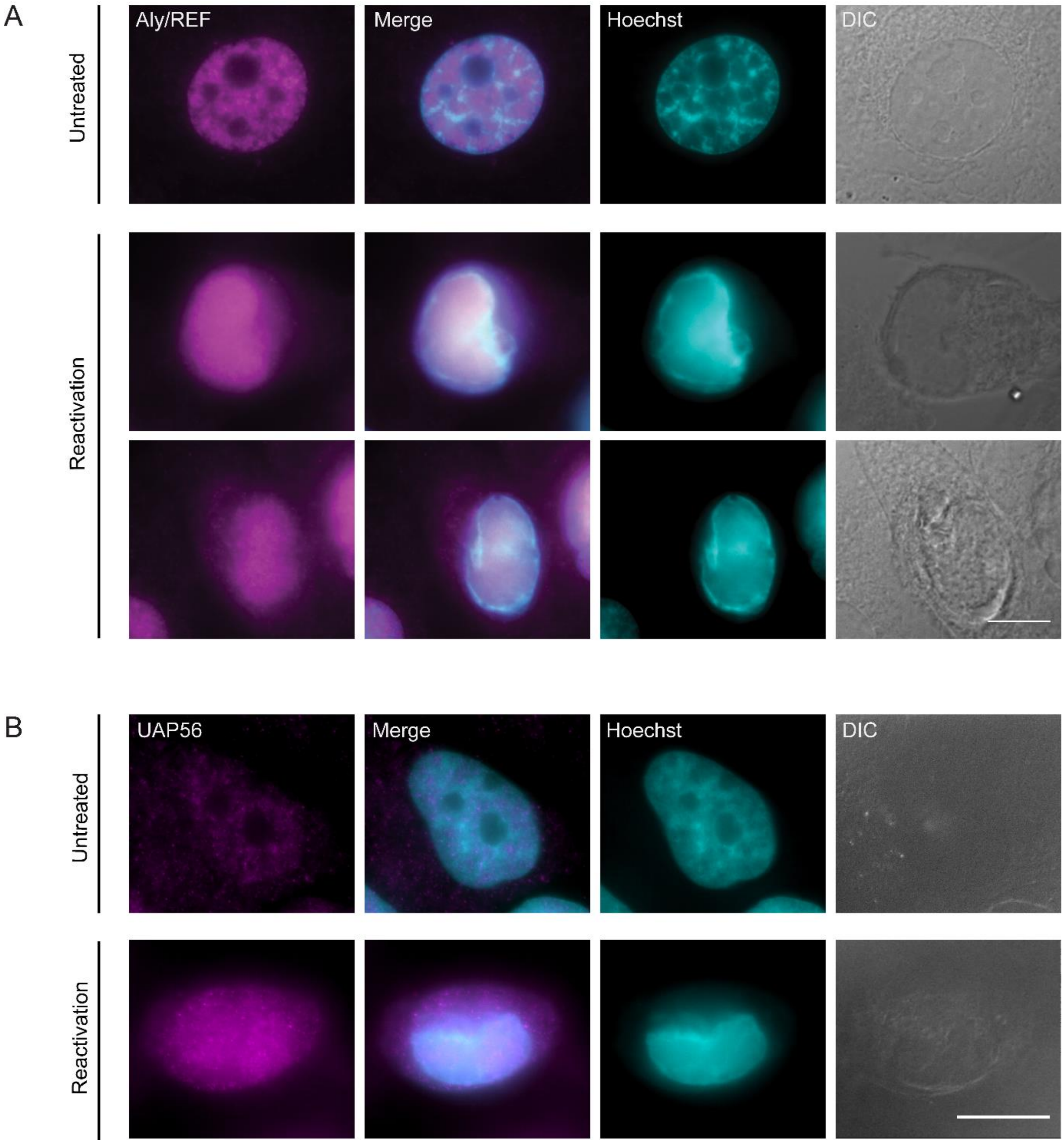

Figure S6. The distribution of Aly/REF and UAP56 during lytic reactivation of KSHV infection. Untreated and BAC16 -mCherry-ORF45-infected iSLK cells induced to the lytic cycle of KSHV infection for 48 hrs and stained with (**A**) anti-Aly/REF or (**B**) anti-UAP56 (magenta); Hoechst in cyan; DIC in grey. Bar, 10 µm.

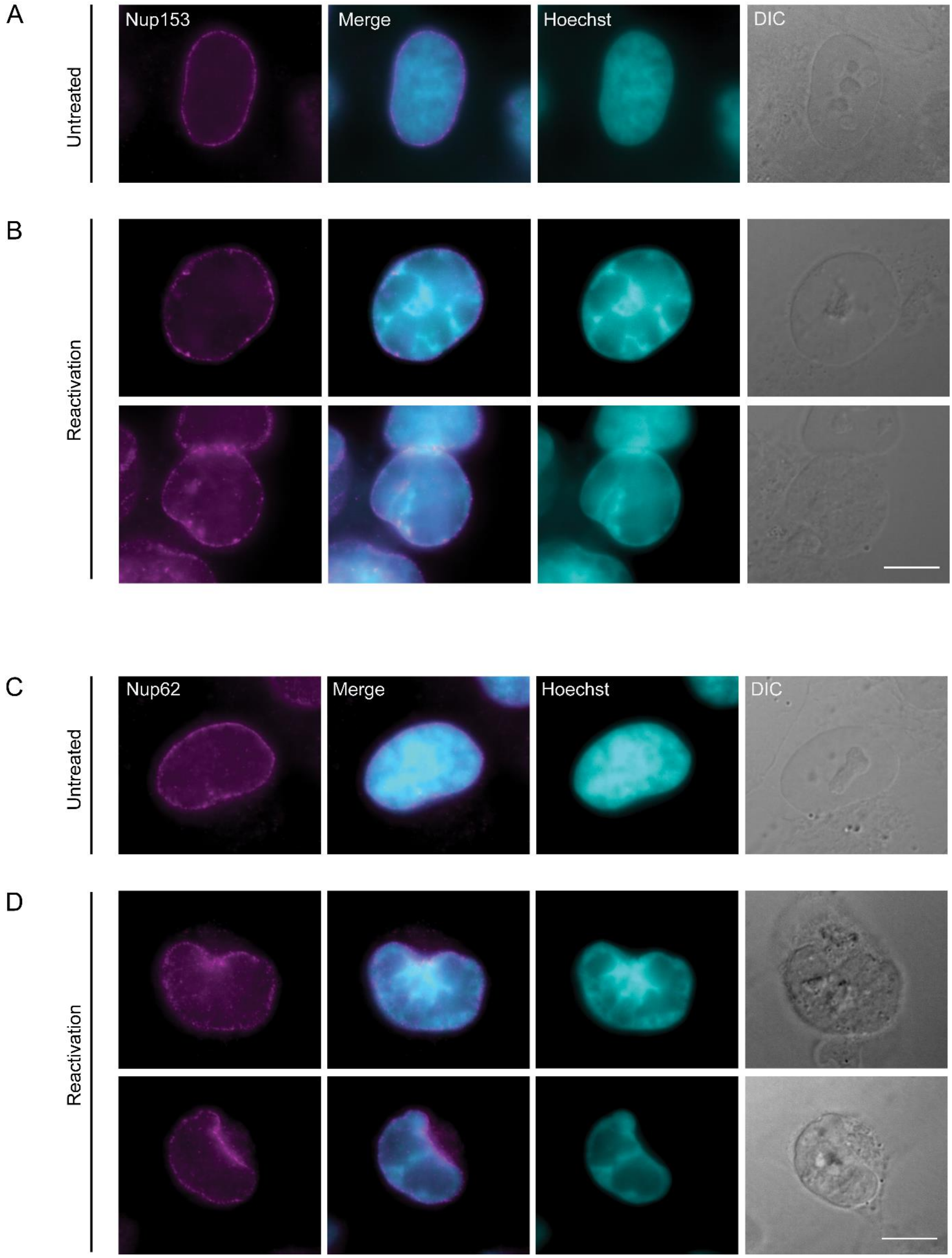

Figure S7. The distribution of nucleoporins during lytic reactivation of KSHV infection. Untreated and BAC16 -mCherry-ORF45-infected iSLK cells induced to the lytic cycle of KSHV infection for 48 hrs and

stained with (**A,B**) anti-Nup153 or (**C,D**) anti-Nup62 (magenta); Hoechst in cyan; DIC in grey. Bar, 10 μm.

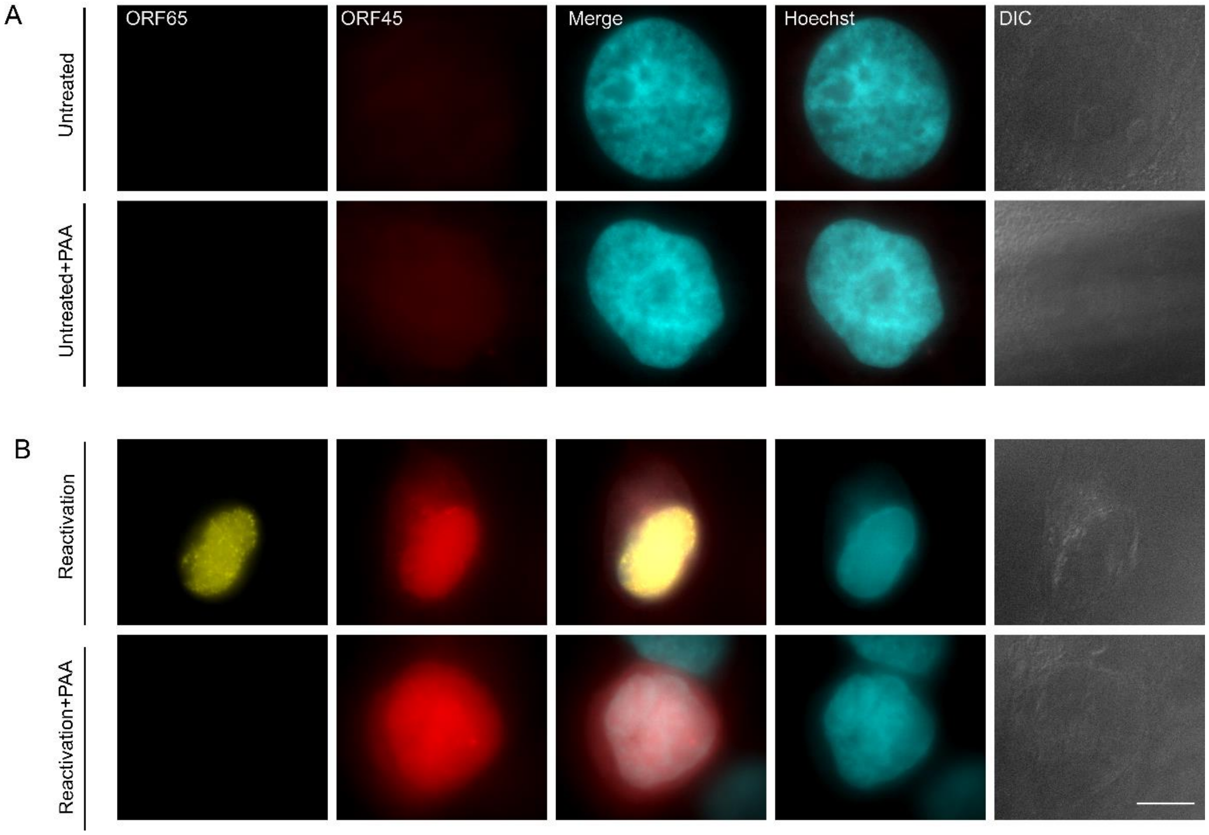

Figure S8. PAA stops the late phase of the lytic cycle. (**A**) Addition of PPA to control uninduced cells did not cause any changes. There was no ORF45 expression (red) indicating that the virus was not induced, nor ORF65 expression (yellow) that is a marker for late lytic gene expression. (**B**) Reactivation conditions induce the expression of both ORF65 and ORF45 but when PAA was added there was no expression of the late marker ORF65. Hoechst in cyan; DIC in grey. Bar, 10 µm.

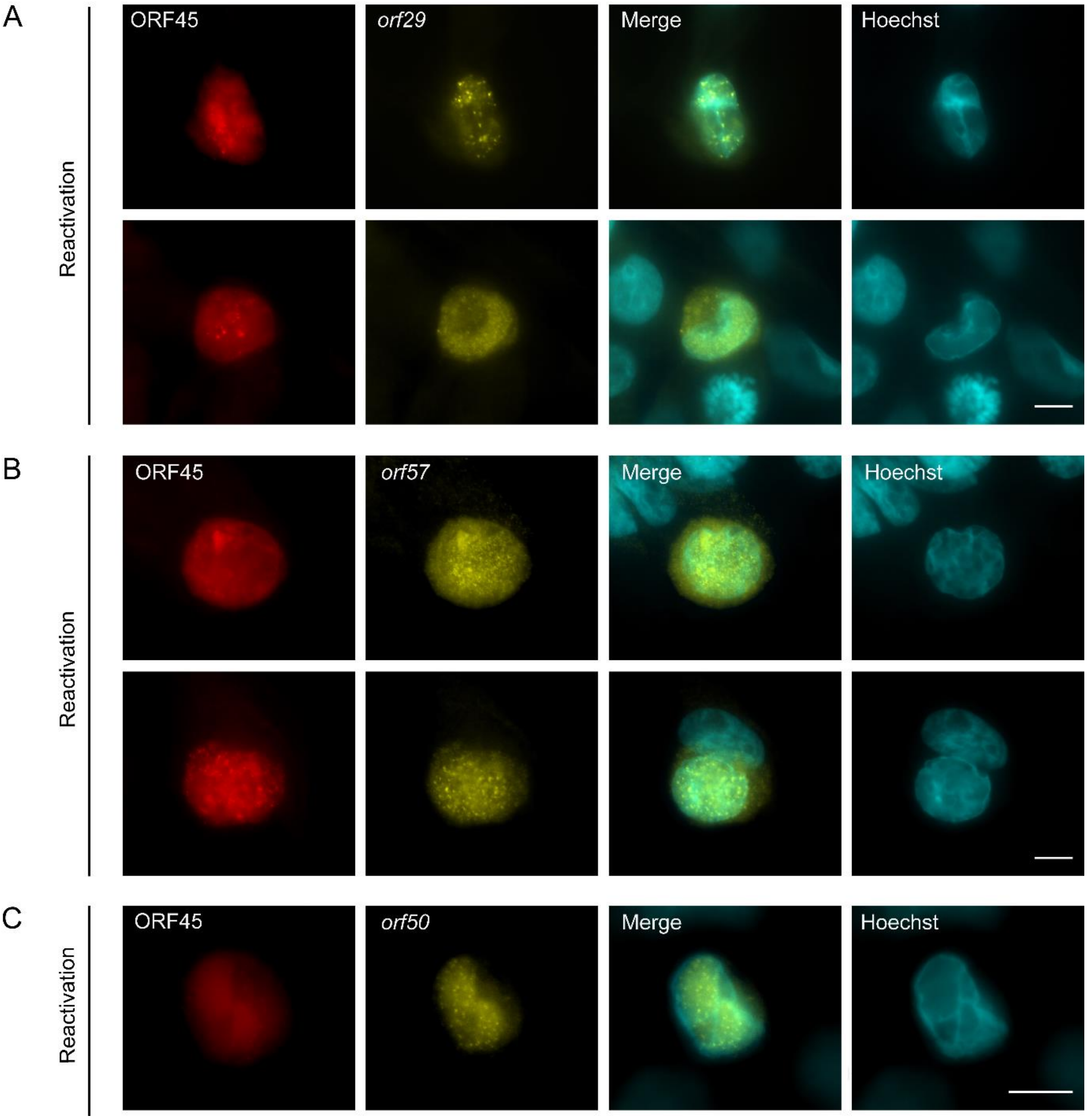

Figure S9. The distribution of viral mRNAs during lytic reactivation of KSHV infection. (**A**) *orf29*, (**B**) *orf57* and (**C**) *orf50* mRNAs (yellow) were detected by RNA FISH in BAC16 -mCherry-ORF45-infected iSLK cells induced to the lytic phase of KSHV infection for 48 hrs. ORF45 in red; Hoechst in cyan; DIC in grey. Bar, 10 µm.

Supplemental Movie Legends

Movie S1. The distribution of nuclear speckles in KSHV infected cells. 3D stacks of infected cells (treated for 72 hrs to induce lytic reactivation) stained with anti-SRSF2 (magenta) and Hoechst (cyan). Each z-stack contains 31 planes at 0.5 μm steps.

Movie S2. The distribution of nuclear speckles in KSHV infected cells. 3D stacks of infected cells (treated for 72 hrs to induce lytic reactivation) stained with anti-SRSF2 (magenta) and Hoechst (cyan). Each z-stack contains 31 planes at 0.5 μm steps.

Movie S3. The distribution of nuclear speckles and RNA Pol II in KSHV infected cells. 3D stacks of infected cells (treated for 72 hrs to induce lytic reactivation) stained with anti-SRSF2 (magenta), anti-RNA Pol II (yellow) and Hoechst (blue). Each z-stack contains 21 planes at 0.5 μm steps.

Movie S4. The distribution of nuclear speckles and RNA Pol II in KSHV infected cells. 3D stacks of infected cells (treated for 72 hrs to induce lytic reactivation) stained with anti-SRSF2 (magenta), anti-RNA Pol II (yellow) and Hoechst (blue). Each z-stack contains 21 planes at 0.5 μm steps.
